# Birth of dairy 4.0: Opportunities and challenges in adoption of fourth industrial revolution technologies in the production of milk and its derivatives

**DOI:** 10.1016/j.crfs.2023.100535

**Published:** 2023-06-24

**Authors:** Abdo Hassoun, Guillermo Garcia-Garcia, Hana Trollman, Sandeep Jagtap, Carlos Parra-López, Janna Cropotova, Zuhaib Bhat, Piera Centobelli, Abderrahmane Aït-Kaddour

**Affiliations:** a Univ. Littoral Côte D’Opale, UMRt 1158 BioEcoAgro, USC ANSES, INRAe, Univ. Artois, Univ. Lille, Univ. Picardie Jules Verne, Univ. Liège, Junia, F-62200, Boulogne-sur-Mer, France; b Sustainable AgriFoodtech Innovation & Research (SAFIR), F-62000, Arras, France; c Department of Agrifood System Economics, Centre ‘Camino de Purchil’, Institute of Agricultural and Fisheries Research and Training (IFAPA), P.O. Box 2027, 18080, Granada, Spain; d School of Business, University of Leicester, Leicester, LE2 1RQ, UK; e Sustainable Manufacturing Systems Centre, School of Aerospace, Transport & Manufacturing, Cranfield University, Cranfield, MK43 0AL, UK; f Department of Biological Sciences, Ålesund, Norwegian University of Science and Technology, Larsgårdsvegen 4, 6025, Ålesund, Norway; g Division of LPT, SKUAST-J, J&K, India; h Department of Industrial Engineering, University of Naples Federico II, P.le Tecchio 80, 80125, Naples, Italy; i Université Clermont Auvergne, INRAE, VetAgro Sup, UMRF, F-63370, Lempdes, France

**Keywords:** Dairies, Industry 4.0, Automation, Smart factory, Real-time monitoring, Artificial intelligence, Big data, IoT, Robotics, Digital technology, 3D printing, Blockchain, Milk, Cheese

## Abstract

Embracing innovation and emerging technologies is becoming increasingly important to address the current global challenges facing many food industry sectors, including the dairy industry. Growing literature shows that the adoption of technologies of the fourth industrial revolution (named Industry 4.0) has promising potential to bring about breakthroughs and new insights and unlock advancement opportunities in many areas of the food manufacturing sector. This article discusses the current knowledge and recent trends and progress on the application of Industry 4.0 innovations in the dairy industry. First, the “Dairy 4.0” concept, inspired by Industry 4.0, is introduced and its enabling technologies are determined. Second, relevant examples of the use of Dairy 4.0 technologies in milk and its derived products are presented. Finally, conclusions and future perspectives are given. The results revealed that robotics, 3D printing, Artificial Intelligence, the Internet of Things, Big Data, and blockchain are the main enabling technologies of Dairy 4.0. These advanced technologies are being progressively adopted in the dairy sector, from farm to table, making significant and profound changes in the production of milk, cheese, and other dairy products. It is expected that, in the near future, new digital innovations will emerge, and greater implementations of Dairy 4.0 technologies is likely to be achieved, leading to more automation and optimization of this dynamic food sector.

## Highlights

• Advanced technologies are being progressively adopted in the dairy sector, from farm to table.
• Dairy 4.0 is mainly enabled by robotics, Artificial Intelligence, Internet of Things, Big Data, and Blockchain.
• Advanced technological solutions proposed for a more sustainable milk production.
• Full implementation of Industry 4.0 technologies in the dairy sector will significantly improve its automation.

## Graphical abstract

**Figure:**
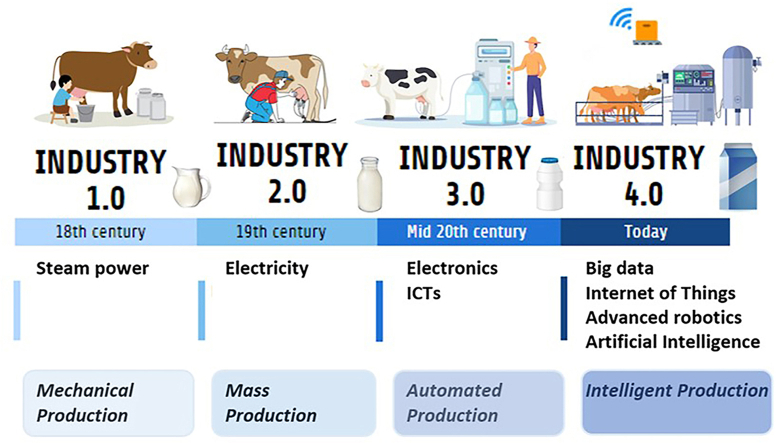

## 1 Introduction

Food sustainability is currently facing several unprecedented crises all at once, including climate change, outbreak of pandemics, and political tensions, among others. Building resilient food systems, and encouraging innovation and implementation of technological advances are of utmost importance to meet these challenges (Bisoffi et al., 2021; Hassoun, Siddiqui, et al., 2022a; Galanakis, 2023). One food sector that is highly vulnerable to climate change and other global food challenges, but also is responsible for a significant release of greenhouse gas emissions, is the dairy industry (Ioanna et al., 2022).

Dairy foods are considered an important source of many nutritional and functional compounds. Critical operating conditions for process control of dairy products have been widely studied to ensure high quality and safety of these highly perishable food products (Neokleous et al., 2022; Ribeiro et al., 2022). However, dairy product manufacturers are always searching for sustainable processing techniques and other new technologies in order to offer innovative solutions to this dynamic food sector. The dairy industry actors are among the fast adopters of new technologies and automation spurred by the arrival of the fourth industrial revolution (namely Industry 4.0). The main enablers of Industry 4.0 in the food sector are Artificial Intelligence (AI), robotics, smart sensors, 3D printing, the Internet of Things (IoT), Big Data (BD), the cloud, blockchain, augmented reality, cybersecurity, digital twins, and cyber physical systems (Trivelli et al., 2019; Bai et al., 2020; Hassoun, Aït-kaddour et al., 2022c). Application of these technologies in agriculture is often referred to as smart farming, precision agriculture, or Agriculture 4.0 (Javaid et al., 2022; Sinha and Dhanalakshmi, 2022), while food factories that use these advanced technologies are often called “smart factories” (Echegaray et al., 2022; Mavani et al., 2022).

The dairy supply chain is increasingly adopting automation and digital technologies (Echegaray et al., 2022; Balaska et al., 2023). The application of Industry 4.0 technologies in the dairy sector is termed as “Dairy 4.0”. Although the term Dairy 4.0 was mentioned in two recent publications (Gehlot et al., 2022; Balaska et al., 2023), no references to its definition or enabling technologies were provided. Therefore, this work aims to investigate the potential of Industry 4.0 in the dairy sector, focusing mainly on the latest advances and applications. The organization of the study is as follows: Section 2 describes the methodology used in this study and presents descriptive statistics of the literature review carried out, Section 3 introduces the concept of Dairy 4.0 and determines its enabling technologies, Section 4 discuses some relevant examples of applications of Dairy 4.0 technologies in milk, cheese, and other dairy products, and Section 5 covers the current challenges and future perspectives.

## 2 Literature review and research methodology

A sample of publications investigating the application of Industry 4.0 technologies in the dairy industry was extracted from Scopus (www.scopus.com) database. Scopus was selected in this study as it is the most comprehensive database of abstracts and citations of peer-reviewed literature, offering wide coverage of scientific production in several scientific disciplines, such as science, technology, medicine, and social sciences, among others (Rejeb et al., 2021). The query used was: (TITLE (dairy AND product) OR TITLE (dairies) OR TITLE (milk) OR TITLE (cheese) OR TITLE (butter) OR TITLE (yogurt) AND TITLE (Industry 4.0) OR TITLE (fourth AND industrial AND revolution) OR TITLE (digitalization) OR TITLE (digital AND transformation) OR TITLE (digital AND technologies) OR TITLE (artificial AND intelligence) OR TITLE (big AND data) OR TITLE (Internet AND of AND Things) OR TITLE (smart AND sensors) OR TITLE (blockchain) OR TITLE (3D AND printing) OR TITLE (cloud AND computing) OR TITLE (cyber AND physical AND systems) OR TITLE (robotics) OR TITLE (augmented AND reality)). The choice of these keywords was based on recent literature on application of Industry 4.0 in agriculture and the food industry sector (Trivelli et al., 2019; Hassoun, Abdullah, et al., 2022b; Hassoun, Aït-kaddour et al., 2022c; Abbate et al., 2023; Bigliardi et al., 2023). By including all types of publications (i.e., articles, reviews, conference papers, and book chapters) without setting a time limitation, the search resulted in 151 documents, while limiting the search to English articles resulted in 147 publications.

**Fig. 1:**
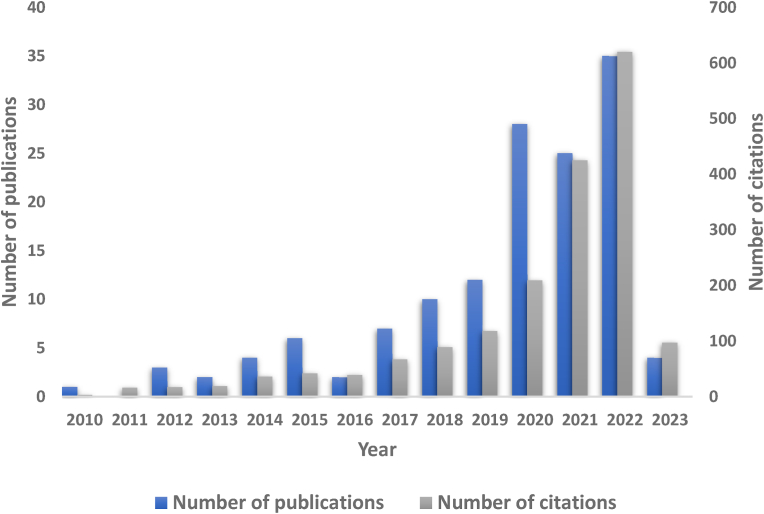
Total publication and citations related to Industry 4.0.

Fig. 1 shows the distribution of research attention in this Industry 4.0 and related topics for each publication year. As can be observed, there is a consistent increase in the number of publications and citations over the last few years, especially since 2015, suggesting that the increasing interest in this topic should become even more relevant in the future. When the year range was limited from 2017 to present, 117 publications were identified and investigated for further analysis.

Table 1 shows the publications on the topic of Dairy 4.0 that have been cited more than 30 times. The subjects of those publications cover almost all the areas of Industry 4.0 (i.e., BD, 3D printing, blockchain, AI, and digital technology). Three of the highly cited publications are related to BD, in which one publication by Schneider et al. (2016) has the highest citation number (229 citations). The publications related to 3D printing have the second highest number of citations. The list of highly cited publications related to Industry 4.0 also shows two publications focused on blockchain and two publications related to AI. This overview shows that the citation number of these topics is increasing rapidly, highlighting the high interest of the dairy industry and scientists in Industry 4.0 and related technologies.

**Table 1:** The most cited publications focusing on Industry 4.0 in the dairy sector.

Authors and Publication Year	Title	Industry 4.0 Technology	Journal	Citation on Scopus
Schneider et al. (2016)	Big Data from Pharmaceutical Patents: A Computational Analysis of Medicinal Chemists Bread and Butter	Big Data	Journal of Medicinal Chemistry	229
Le Tohic et al. (2018)	Effect of 3D printing on the structure and textural properties of processed cheese	3D printing	Journal of Food Engineering	173
Liu et al. (2019)	Rheological and mechanical behaviour of milk protein composite gel for extrusion-based 3D food printing	3D printing	LWT	105
Liu et al. (2018)	3D printed milk protein food simulant: Improving the printing performance of milk protein concentration by incorporating whey protein isolate	3D printing	Innovative Food Science and Emerging Technologies	84
Casino et al. (2021)	Blockchain-based food supply chain traceability: a case study in the dairy sector	Blockchain	International Journal of Production Research	78
Goli et al. (2019)	Hybrid artificial intelligence and robust optimization for a multi-objective product portfolio problem Case study: The dairy products industry	Artificial Intelligence	Computers and Industrial Engineering	55
Goli et al. (2021)	An integrated approach based on artificial intelligence and novel meta-heuristic algorithms to predict demand for dairy products: a case study	Artificial Intelligence	Network: Computation in Neural Systems	42
Mangla et al. (2021)	Using system dynamics to analyze the societal impacts of blockchain technology in milk supply chainsrefer	Blockchain	Transportation Research Part E: Logistics and Transportation Review	35
(Newton et al., 2020)	Farming smarter with big data: Insights from the case of Australia's national dairy herd milk recording scheme	Big Data	Agricultural Systems	33
Drewry et al. (2019)	Assessment of digital technology adoption and access barriers among crop, dairy and livestock producers in Wisconsin	Digital technology	Computers and Electronics in Agriculture	32
Cabrera et al. (2020)	Symposium review: Real-time continuous decision making using big data on dairy farms	Big Data	Journal of Dairy Science	31

**Fig. 2:**
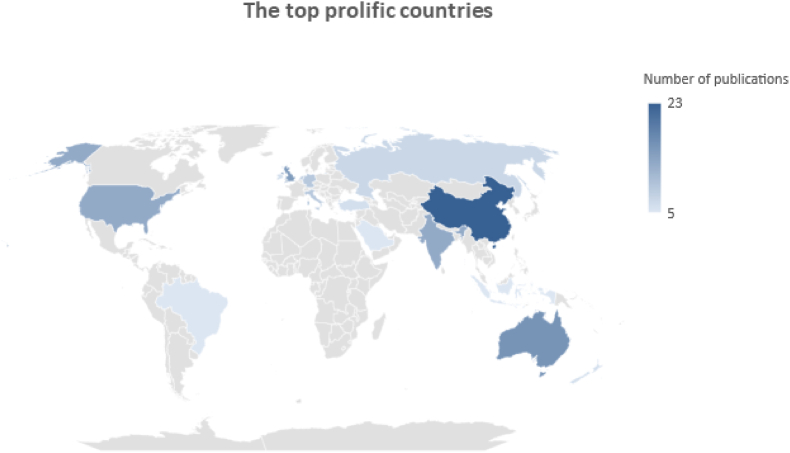
Top contributing countries to publications related to Industry 4.0.

Fig. 2 shows the top contributing countries to publications dealing with the topic of Industry 4.0 and related technologies in the dairy sector. A total of 15 countries participated in publishing of at least 5 or more publications. China was on the top of the rank with 23 publications, followed by Australia (16 publications), UK (15 publications), India (13 publications) and USA (13 publications), highlighting the importance of the dairy sector in these countries.

**Fig. 3:**
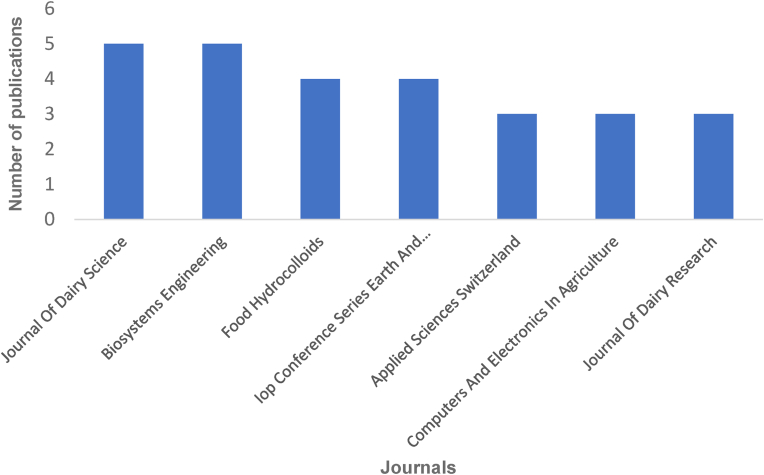
Most prominent sources by the number of publications in this field of Industry 4.0 in the dairy sector.

Regarding the document types, 64% of the total number of publications were articles, while conference papers represented 26%. As reported in Fig. 3, only seven journals have published three or more papers. The highest number of publications (five publications) was found in the Journal of Dairy Science and Biosystems Engineering.

**Fig. 4:**
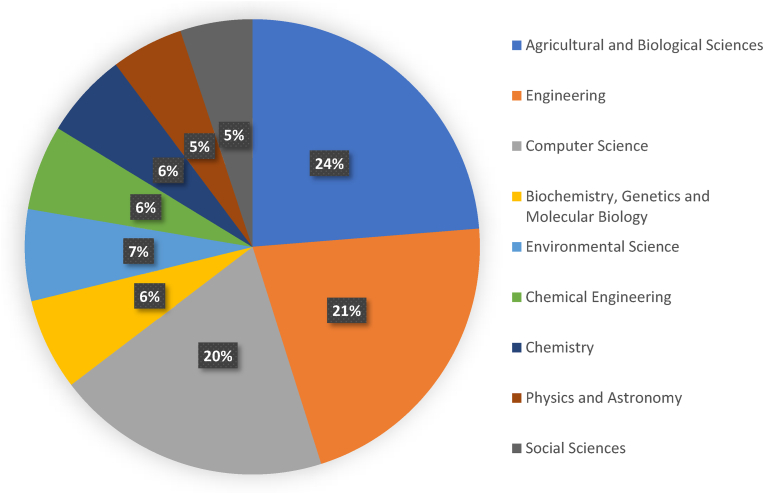
Publication related to Industry 4.0 in the dairy sector according to their subject areas.

Fig. 4 provides the percentage of publications for each subject area of Industry 4.0. The most prominent areas are agricultural and biological sciences, engineering, and computer science that exhibited about two-thirds of the total number of publications, while physics and astronomy and social science areas had the fewest number of publications (only 5% of the total) in relation to Industry 4.0.

## 3 Dairy 4.0 enabling technologies

**Fig. 5:**
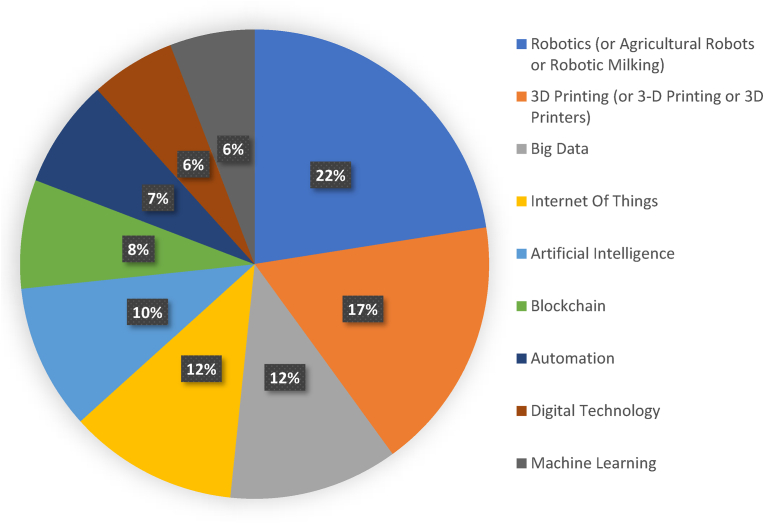
Most used keywords in published research related to Industry 4.0 in the dairy sector.

Our literature review revealed that robotics, 3D printing, AI, IoT, BD, and blockchain are the most keywords used in the published research related to Industry 4.0 technologies and their application in the dairy sector (Fig. 5).

**Fig. 6:**
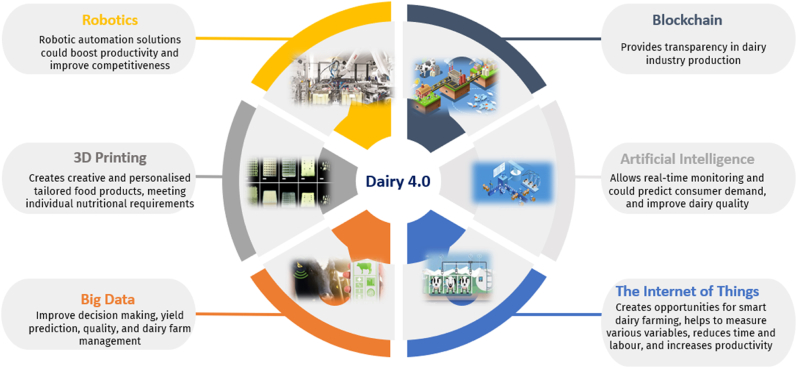
Dairy 4.0 enabling technologies.

Six Industry 4.0 technologies have been identified and considered the main enablers of Dairy 4.0 (Fig. 6). These Dairy 4.0 will be briefly discussed in this section:

**Robotics** – Robotics is an interdisciplinary and multidisciplinary field that combines advanced systems related to mechanical, electrical and electronics hardware and software. The use of robotics in the food industry can vary between simple repetitive tasks to complex processes. Robotics technology has been long in use in many manufacturing sectors, e.g., automotive, but potential applications for robotics in dairy industries is being investigated. (Sandey et al., 2017; Gehlot et al., 2022).

**3D Printing** – Three-dimensional (3D) printing technology (or additive manufacturing) has emerged recently as a promising innovation that use digital data to create various types of solid and semi-solid food products (Zhang et al., 2022). Over the last few years, the application of 3D printing has gained enormous interest in many food sectors (Hu et al., 2022). There has been a rapid increase in scientific work addressing 3D printing of dairy products. Dairy products and ingredients consists of different constituents, functionalities and structures making them a very challenging but at the same time very promising source of raw materials for 3D printing of food (Lee et al., 2020; Le Tohic et al., 2018).

**Big Data (BD)** - BD refers to extremely large and complex data sets that cannot be analysed or processed using traditional methods (Ayed et al., 2022). Big Data Analytics (BDA) is the process of using advanced analytical tools and techniques to process these massive data sets (Ciccullo et al., 2022). In the dairy industry, BD/BDA can be applied in a number of ways as the dairy industry generates huge amounts of data. Applications relate to milk production, milk quality, farm management, real-time monitoring and decision making, animal health and supply chain sustainability.

**The Internet of Things (IoT)** – IoT is a system that connects devices through a network allowing the exchange of data. IoT has the potential to create intelligent environments of powerful tools in many agriculture and livestock areas, enabling to create, modify, and share information (da Rosa Righi et al., 2020; Karouani and Elgarej, 2022). IoT is increasingly being used in dairies to collect live, accurate data and share it with the farmer or other stakeholders.

**Artificial Intelligence (AI) –** AI is a computational technology used to mimic human abilities to perceive their environment, learn, and make decisions (Manning et al., 2022; Ramirez-Asis et al., 2022). In the food sector, AI techniques play a key role in automation in smart farming and food processing industry and could help to accelerate the transition towards sustainable food systems (Kumar et al., 2021; Marvin et al., 2022). Generally, AI-based technologies can be used in real-time monitoring and decision-making process (Nogoy et al., 2021; Sharma et al., 2021).

**Blockchain** – Blockchain can be considered as one of the Industry 4.0 enablers and can address the growing industry need for considerably more reliable and efficient supply chain management infrastructure (Hassoun, Abdullah, et al., 2022b; Al Nuaimi et al., 2023; Hassoun et al., 2023). Blockchain technology can be defined as cryptographically secured distributed ledgers where the ledgers are distributed in a peer-to-peer format among the entities, making it a decentralized system (Gehlot et al., 2022; Yadav et al., 2022). There is a need for decentralized solutions such as blockchain due to the single points of failure, product irregularities, quality compromises, and loss of data present in conventional food supply chains. Adoption of blockchain in the food supply chain, especially when combined with other technologies such as IoT, enables data interoperability, cost reduction, transparency, auditability, integrity and authenticity (Casino et al., 2021; Luo et al., 2022). To protect vulnerable populations, blockchain solutions that go beyond food traceability to maintaining the nutritional values of dairy products, and identification of their adulteration and contamination have been proposed (Khanna et al., 2022).

## 4 Discussion of applications of dairy 4.0 technologies in milk and dairy products

### 4.1 Robotics

Robotics has been more recently embraced by the dairy sector, where it has several applications. For instance, automatic milking systems are one of the most successful application of robotics in the dairy industry (Sandey et al., 2017; Perov, 2022). This system allows for a substantial average increase in milking frequency and therefore increased milk production (Hogenboom et al., 2019), while reducing labour costs. Further identified benefits of automatic milking systems are increased overall productivity, profitability, and sustainability (Lyons et al., 2022). Another application of robotics in dairy farms is on floor cleaning, which helps to mitigate risks regarding cow welfare and to avoid ammonia emissions (Gerrits et al., 2023).

In addition, sensors placed in the robotic system allow accurate and live data to be collected. These data can be used to predict the value of important parameters such as daily milk yield, milk composition and milking frequency. For instance, Ji et al. (2022) developed a machine learning framework using data from a robotic dairy farm to predict such parameters.

An important concern for dairy farmers for a more widespread adoption of robotic milking systems is the impact these systems might have on the final product. Johansson et al. (2017) investigated the composition and enzymatic activity in milk from dairy farms with conventional and robotic milking systems, and found some differences, suggesting a significant influence of the management system. For instance, milk from robotic milking systems had a lower protein content, plasmin, plasminogen, total plasmin/plasminogen derived activities, total casein and the β-casein fraction as percentage of total protein, while it had a higher somatic cell count and total proteolysis. Another factor analysed in automatic robotic dairies is heat stress, i.e. the effect of high temperature and humidity on the cow welfare and performance. Bodo et al. (2022) and Osei-Amponsah et al. (2020) found a positive correlation between the heat stress and a reduction in milking time and time spent in the milking box, and in general cows’ welfare. Therefore, the performance of robotic milking systems could easily be improved by simply providing shade and water for cows to cool down during hot months. Finally, robotic milking systems also facilitate the work of farmers, reducing their stress and anxiety and increasing their resilience (King et al., 2021).

Despite their benefits, the development and implementation of such robotic systems is challenging. To facilitate wider adoption of robotic systems on dairy farms, Eastwood et al. (2022) proposed a framework to identify issues that technology developers and policy makers need to consider when considering future innovations for robotics on dairy farms, such as the impact on job design, worker welfare and safety, changes in farming systems, and the influences of market and regulatory constraints.

### 4.2 3D printing

Over the last few years 3D printing of milk-based products are gaining popularity among researchers as well as food manufacturers. The 3D printing in dairy is being pursued within 5 thematic areas, i.e., Futuristic, Creative, Healthy, Efficient and Sustainable as shown in Table 2 (Ross et al., 2018).

**Table 2:** Themes with their respective 3D printing applications.

Themes	3D Application
Futuristic	It creates food products with similar consistency and repetition with parameters such as shapes, size and decoration, e.g., celebration cakes artistic decoration.
Creative	It creates creative and personalised specially textured food products to suit individual nutritional requirements without discomfort.
Healthy	It facilitates the accurate dispensing of ingredients/nutrients during printing of food products.
Efficient	It allows layer-by-layer deposition during a process using the most efficient pathway.
Sustainable	It results in zero waste or minimal excess material being disposed of in the process and therefore sustainable.

Liu et al. (2018) showed that the milk powder paste mixture prepared with milk protein concentrate and whey protein isolate (mixed in a certain ratio) was the most desirable material for extrusion-based 3D printing. They researched further to show that the milk protein gel had the best 3D printing performance, which matched the model best and had good fidelity (Liu et al., 2019) and also investigated the effects of hydrocolloids on the microstructures, viscoelastic characteristics and 3D printing performance of milk protein concentrate (Liu et al., 2022).

Some researchers formulated soy protein- and gluten-based gel materials for 3D printing by employing thermosensitive cocoa butter to study its likelihood for preparing meat analogues (Wang and Liu, 2021). (Shahbazi et al., 2021) developed an application of a reduced fat casein-based Pickering emulsions in the 3D printing process to develop functional foods, broadening the micro-biosurfactant utilization in food printing. Similarly (Daffner et al., 2021), presented design and characterisation of casein−whey protein suspensions mixed with dairy fat processed via the pH−temperature-route in preparation for 3D printing. Some researchers showed that the rennet-induced gelation of milk proteins as a potential method for the formation of 3D printed food structures (Uribe-Alvarez et al., 2021), while others investigated 3D printability of the functionalized yogurts on bread, and developed yogurts with bioactive ingredients of high quality and application value (Hu et al., 2022). Li et al. (2022) developed a method for rapid determination of lactose in milk using a bioactive paper-3D printing integration technology.

Lee et al. (2020) developed direct ink writing technique for 3D printing of milk products at room temperature by changing the rheological properties of the printing ink. Le Tohic et al. (2018) used processed cheese as the printing material both at low or high extrusion rates.

### 4.3 Big Data

An important application of BD/BDA is to improve on-farm decision making, milk yield prediction, milk quality improvement and dairy farm management. In this context, Yan et al. (2015) introduced the Milk Yield Prediction and Analysis Tool (PAT), a cost-effective tool that uses BDA to accurately predict milk yield at both individual cow and group levels. This study highlighted the importance of data-driven decision making for small-scale milk producers. Another study by Zhang et al. (2021) discussed the application of 5G + IoT technology, BD mining and analytics, and AI in smart dairy pasture management, specifically to identify individual cows and accurately feed dairy cows. By using image recognition technology, the study showed that this approach can effectively improve the economic benefits and production efficiency of the cattle farm. Recently, Rodriguez-Venegas et al. (2022) investigated the impact of heat stress on milk production using the Temperature Humidity Index (THI) as a biomarker of heat stress, i.e., a THI-BD approach. The study found that heat stress can lead to a reduction in milk production and highlights the need for mitigation strategies to counteract heat stress. Duruz et al. (2020) investigated the impact of transhumance on cattle productivity. The study used big dairy data to develop a model that considers environmental, physiological and morphological factors on milk production during transhumance. Boiarkina et al. (2018) showed that the application of BD in milk powder processing can improve product quality and reduce in off-specification products. According to Newton et al. (2020), the transition to a new business stage, the importance of the whole farm context, and the use of data beyond short-term decision making are important dimensions influencing farmers' demand for and engagement with BD applications to improve farm decision making.

The integration of BD/BDA in dairy farming has shown great potential to improve animal health and productivity, and ultimately dairy farm profitability through strategic management. For example, Cabrera et al. (2020) presented a decision-making engine (The Dairy Brain) that integrates precision agriculture, BDA and IoT. The system collects, integrates, manages, and analyses on- and off-farm data in real-time to provide practical applications such as nutrient grouping, early risk detection of clinical mastitis, and prediction of clinical mastitis onset. The results show that the system has the potential to reduce nutritional costs and improve health monitoring. Animal health assessment is an important research area for the use of BD. Assessing the global health of animals is a complex task due to its multifaceted nature, and researchers tend to focus on detecting specific diseases separately. In this context, Franceschini et al. (2022) proposed an approach to assess the global health status of dairy cows using BD from milk records, including milk yield, somatic cell count and Fourier transform mid-infrared (FT-MIR)-based predictors related to milk composition and animal health status. The results suggest that the quantitative traits obtained indirectly reflect some of the major health disorders in dairy farming and could be used to monitor dairy cows on a large scale. In another study, Pralle and White (2020) discussed the challenges faced by dairy farms in detecting hyperketonemia (HYK), a metabolic disorder affecting cows during the transition to lactation. The results suggested that the increasing data streams available to farms, such as milk production and composition, cow management records and genomics, can be used to monitor the onset of postpartum HYK using a BD approach.

BD technologies can also be used to improve supply chain management. However, there are several potential barriers that need to be addressed for their successful implementation. In this context (Kazancoglu et al., 2021), identified economic as the most important barrier to circularity in dairy supply chains, followed by technological and environmental. Optimization is found to be the most important BD solution to overcome these barriers, followed by data mining and machine learning. The main applications of BD/BDA identified are in line with the findings of (Lokhorst et al., 2019). These authors found that the majority of research in precision dairy farming focuses on the animal sublevel, with topics at the dairy farm level dominating. The authors concluded that the full potential of BD in precision dairy farming has not yet been realised and that multiple BD characteristics and sources need to be used to add value to decision making.

### 4.4 Internet of Things

There are many types of data that IoT can collect and share, such as temperature, heart rate and cow movement, which provide information on the health, position and behaviour of the cows, such as standing, lying down, eating or oestrus events (Pratama et al., 2019; Arago et al., 2022). These data can help farmers to make more informed decisions, for instance regarding the cow health, feeding and milking frequency.

IoT has great potential to collect and share live data from dairies. In fact, recent research has suggested the use of IoT to automate cow feeding, so that nutrition plans can be designed for each cow and a more accurate milk production forecast can be made for each cow (da Rosa Righi et al., 2020). Yavari et al. (2020) also proposed a low-cost IoT solution for milk-quality monitoring. Faruq et al. (2019), on the other hand, used IoT to monitor the temperature and heart rate data of cows, which provides information about the cow's health and enables disease diagnosis and the identification of the most adequate treatment and prevention method.

The potential of IoT extends beyond only the farm to the entire dairy supply chain. There are several examples of using IoT to optimise the logistics of dairy products (e.g., Zou et al., 2020; Kazancoglu et al., 2022; Chakurkar et al., 2018; Karouani and Elgarej, 2022). In the dairy industry, logistics is an important aspect due to the high perishability of dairy products and the need to distribute these products to all population centres.

Finally, IoT can also help to measure the value of different variables that affect the quality of fresh milk, such as pH, temperature, odour, turbidity, colour, fat, taste and the presence of additives (Habsari et al., 2022; Chen et al., 2022). This helps to classify the milk according to its quality and thus determine its optimal price.

### 4.5 Artificial Intelligence

AI has been mainly employed to assess the health and wellbeing of dairy cows. This is due to a significant need within the dairy industry for robust, automated, and inexpensive technology for the analysis of dairy cow behaviour (Lima et al., 2021). AI can mine data from various currently underused sources to simplify repetitive and difficult decision-making tasks in dairy farming (Nogoy et al., 2021). AI selects the optimal treatment of cows based on the impacts of physiological and environmental factors for superior milk quality and production (Sugiono et al., 2017). Other applications of AI in Dairy 4.0 include consumer demand, food quality, and dairy farming techniques.

The prediction of demand is very important in the dairy industry as most products have a short shelf-life. The implementation of AI has been used to calculate product portfolio risk (Goli et al., 2019) and for demand prediction (Goli et al., 2020, 2021) with application to the dairy industry of Iran.

Food quality is increasingly important to consumers, yet few tools exist outside of advanced laboratories, and current inspection mechanisms are unable to adequately process the various threats to food integrity. Sound vibrations traversing food products used in conjunction with AI have been proposed to detect adulteration and verify quality (Iymen et al., 2020). An optimization process based on AI has been proposed to identify sensory improvements to food products (Bi et al., 2022). Computer vision and machine learning techniques have been used to determine cheese quality during the ripening process (Loddo et al., 2022). The heatwaves resulting from climate change are stressing farm animals. A system based on AI has been proposed to increase or maintain milk quality by reducing heat stress (Fuentes et al., 2020).

There is also pressure to increase herd size while reducing animal husbandry and welfare costs in which AI methods can provide significant support (Hajnal et al., 2022). AI can be used to predict milk production and quality based on computer vision methods that estimate the heart rate and respiration rate of cows (Fuentes et al., 2021). AI has also been applied to aerial imagery to estimate dairy methane emissions and herd size (Jeong et al., 2022).

### 4.6 Blockchain

Blockchain can be utilized for building intelligent ecosystems for milk supply chains to help meet the demand for food of the growing global population in a sustainable manner (Gehlot et al., 2022). Varavallo et al. (2022) proposed a blockchain-based traceability solution for lowered environmental impact and reduced cost per transaction applied to the Fontina PDO cheese supply chain. Mangla et al. (2021) investigated societal impacts of blockchain technology to build social sustainability in milk supply chains in Turkey and found several benefits in terms of food fraud prevention, rural development, animal health and welfare in addition to promoting healthy food and food security.

Makarov et al. (2019) proposed a blockchain solution for the management of dairy supply chain products to ensure the safety of data, protection of repository documents from hacking, immutability of data on the progress of transportation, and to enable both retailers and consumers to investigate supply chain actors from farm to consumer. Casino et al. (2021) focused their blockchain application on proof of regulatory compliance to both state authorities and demanding customers. Fang and Stone (2021) proposed a dairy logistics supply ecosystem with blockchain technology to provide fresh and safe dairy products at fair prices by employing smart contacts with crowdfunding functionality.

## 5 Current challenges and future perspectives

There are still several challenges that the industry must overcome to fully exploit the benefits of Industry 4.0 technologies.

One of the main challenges is related to the territorial location: the lack of digital infrastructure and connectivity in many rural areas where dairy farms are located impede the full spread of technologies. This can make it difficult for farmers to access and use new technologies for the exploitation of precision farming and automated milking systems (Zambon et al., 2019; Dadi et al., 2021). Another challenge is the high cost of implementing new technologies, particularly for SMEs sized dairy farms typically characterized by limited financial resources (Zambon et al., 2019; Bahn et al., 2021). This will lead to an increase in the digital divide between larger and smaller dairy farms in all the countries, with the former having a competitive advantage due to their greater ability to invest in new technologies.

Another issue emerging from the analysis is the need for skilled employees operating in the industry and implementing these new technologies. The companies that want to implement these technologies must equip themselves with skilled human resources and provide necessary tools and equipment for their training. Even in this case, this aspect can be a challenge for smaller farms that may not find skilled resources to hire or take care of the employees’ training. Data privacy and cybersecurity issues are also important concerns for the dairy industry. With the increasing use of connected devices and data analytics in dairy production, there is a risk of sensitive data being compromised (Bahn et al., 2021; Goller et al., 2021).

Finally, also in the dairy industry a successful implementation of Industry 4.0 technologies requires a greater collaboration and coordination among supply chain stakeholders to ensure that new technologies are effectively integrated into existing systems.

In conclusion, even while technology advancements and digitalization have the potential to be very beneficial for the dairy business, there are still a number of obstacles that must be overcome in order to fully realize these advantages. These include issues related to digital infrastructure and connectivity, the cost of implementing new technologies, the need for skilled labour, and the need for greater collaboration among supply chain actors. In order to take advantage of technological advancements and make sure that the dairy business is competitive and sustainable in the future, it will be essential to address these difficulties.

## Credit author statement

Abdo Hassoun: Conceptualization, Methodology / Study design, Resources, Data curation, Writing – original draft, Supervision, Project administration. Guillermo Garcia-Garcia: Methodology / Study design, Resources, Data curation, Writing – original draft, Writing – review & editing. Hana Trollman: Methodology / Study design, Resources, Data curation, Writing – original draft, Writing – review & editing. Sandeep Jagtap: Methodology / Study design, Resources, Data curation, Writing – original draft, Writing – review & editing. Carlos Parra-López: Methodology / Study design, Resources, Data curation, Writing – original draft, Writing – review & editing. Janna Cropotova: Methodology / Study design, Resources, Data curation, Writing – original draft, Writing – review & editing, Supervision, Project administration, Funding acquisition. Zuhaib Bhat: Methodology / Study design, Resources, Data curation, Writing – original draft, Writing – review & editing. Piera Centobelli: Methodology / Study design, Resources, Data curation, Writing – original draft, Writing – review & editing. Abderrahmane Aït-Kaddour: Conceptualization, Methodology / Study design, Resources, Data curation, Writing – original draft, Writing – review & editing, Supervision, Project administration.

## Declaration of competing interest

The authors declare that they have no known competing financial interests or personal relationships that could have appeared to influence the work reported in this paper.

## Data Availability

No data was used for the research described in the article.
